# *Pseudocapillaria tomentosa* (Nematoda: Capillariidae) in fish and its significance in comprehending host-parasite relationships: A review

**DOI:** 10.1016/j.crpvbd.2025.100265

**Published:** 2025-05-06

**Authors:** Amin Marandi, Anne Majgaard Jensen, Louise von Gersdorff Jørgensen

**Affiliations:** a Department of Aquatic Animal Health, Faculty of Veterinary Medicine, University of Tehran, Tehran, Iran; b Experimental Fish Models (Exfimo), Department of Veterinary and Animal Sciences, Faculty of Health and Medical Sciences, University of Copenhagen, Frederiksberg C, DK1870, Denmark

**Keywords:** Capillariids, *Danio rerio*, Biosecurity, Quarantine, *Pseudocapillaria tomentosa*

## Abstract

Helminth parasites, including capillariids, pose a significant health risk to animals, including fishes, birds, and mammals. *Pseudocapillaria tomentosa* is a widespread, but poorly studied parasite primarily infecting freshwater cyprinid fishes in the northern hemisphere. However, despite controlled laboratory procedures, the parasite is also prevalent in many zebrafish (*Danio rerio*) research facilities due to inadequate measures to ensure biological security and the ability of the nematode to endure chlorine treatment. *Pseudocapillaria tomentosa* typically causes chronic disease in fish, leading to morbidity, mortality, and economic challenges. Clinical signs include emaciation, ulcers, anemia, and hemorrhage, as well as reduced growth and reproductive ability, and prominent humpback lesions in severe infections. Diagnosing *P. tomentosa* infections involve observation of eggs in wet mount preparations or worms in histological sections of the intestine. However, definitive species identification requires detailed morphological examination and molecular analyses. *Pseudocapillaria tomentosa* can be effectively managed through anthelmintic treatments such as emamectin, fenbendazole, albendazole, and mebendazole as well as preventive measures including maintaining optimal water quality, reducing fish density, and implementing strict quarantine protocols. This review discusses the use of the zebrafish to study host-parasite interactions, particularly with the parasitic nematode *P. tomentosa*. The zebrafish is a suitable model for studying infectious diseases, including parasites, due to its well-characterized immune system, reporter lines and cell lineages. This model organism exhibits immune responses to helminth antigens, including eosinophilia and the upregulation of inflammatory cytokines like Tnf-α and Ifn-γ. The gut microbiota plays a crucial role in susceptibility to parasitic infections in zebrafish and an imbalanced or dysbiotic gut microbiome can predispose fish to parasitic infections, while a healthy, balanced microbiome can enhance resistance. Furthermore, probiotic-based interventions are being explored as a way to boost mucosal immunity and modify the gut microbiome to prevent parasitic infections.

## Highlights

• The host-parasite relationships of the nematode *Pseudocapillaria tomentosa* are reviewed.
• *P. tomentosa* causes chronic disease and high mortality in fish.
• Diagnosis and species identification require microscopy, detailed morphological and genetic analyses.
• Control measures include dewormers and prevention through water quality, density control, and quarantine.
• Zebrafish is an ideal model organism to study parasites.

## Graphical abstract

**Figure:**
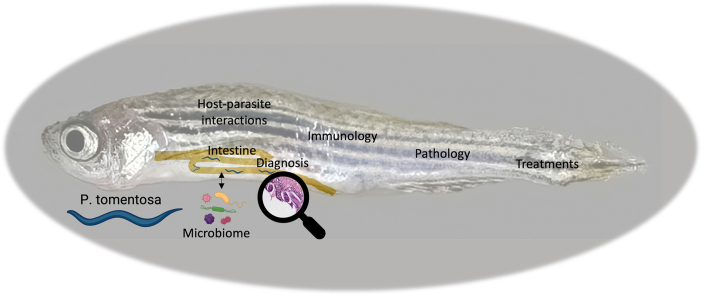

## 1 Introduction

Helminth parasites pose a substantial risk to the health of wild, captive, and research animals (Gaulke et al., 2016). Capillariids, a nematode family within the broader category of helminth parasites, are notable for their ability to infect a wide range of vertebrates, including fishes, birds, and mammals, and for their pathogenicity due to their tissue-invasive nature (Moravec, 1982, 1983; Moravec et al., 1984, 1987; Košuth, 1997; Kent et al., 2002; Rahmati-Holasoo et al., 2022a). Various species of capillariid nematodes are known to cause diseases in fishes, particularly among ornamental species including guppy (*Poecilia reticulata*), angelfish (*Pterophyllum scalare*), tiger barb (*Puntigrus tetrazona*), topmouth gudgeon (*Pseudorasbora parva*), and green terror (*Andinoacara rivulatus*) (Moravec et al., 1999; Mihok et al., 2011; Murray and Peterson, 2015; Kent et al., 2018; Rahmati-Holasoo et al., 2022a, 2022b). Fish capillariids typically demonstrate their specificity by targeting specific host families (Moravec, 2001).

Based on the classification system presented by Moravec (2001), species of the genus *Pseudocapillaria* that infect cold-blooded vertebrates can be categorized into four subgenera, *Discocapillaria*, *Ichthyocapillaria*, *Indocapillaria*, and *Pseudocapillaria*. This classification is determined by examining the morphology of the male caudal end and the presence or absence of a vulval appendage (Moravec and Justine, 2010). *Pseudocapillaria tomentosa* Dujardin 1843 is a parasitic nematode that has been classified into the class Enoplea, order Trichocephalida, and family Capillariidae (Moravec, 1982; Košuth, 1997). *Pseudocapillaria brevispicula* and *Capillaria patzcuarensis* are regarded as senior synonyms of *P. tomentosa* (Moravec et al., 1999; Mejía-Madrid et al., 2005; Kent and Sanders, 2020). This parasite, commonly found in the rectum and posterior sections of the intestine (Mihok et al., 2011), predominantly infects freshwater cyprinid fishes in the northern hemisphere (Moravec, 2001). Furthermore, eels, cod fish, salmon, and catfish in the orders Anguilliformes, Gadiformes, Salmoniformes, and Siluriformes, respectively, are also vulnerable to this infection (Moravec, 1987; Kent et al., 2002). The geographical and anatomical distribution of *P. tomentosa* across various species of fish hosts is provided in Table 1.

**Table 1:** Geographical and anatomical distribution of *Pseudocapillaria tomentosa* in different host fish species.

Host species	Body location	Country	Reference
*Algansea lacustris*	Intestine	Mexico	Pérez-Ponce de León et al. (2000)
*Alloophorus robustus*	Intestine	Mexico	Pérez-Ponce de León et al. (2000)
*Astyanax lacustris*	Intestine	Mexico	Garfias et al. (1996)
*Barbus capito*	Intestine	Iran	Pazooki et al. (2003)
*Barbus lacerta*	Intestine	Iran	Pazooki et al. (2003)
*Barbus tetrazona*	Intestine	Germany	Moravec et al. (1999)
*Carassius gibelio*	Intestine	The Republic of Moldova	Gologan (2020)
*Catostomus commersoni*	Intestine	Mexico	Mejía-Madrid et al. (2005)
*Chirostoma attenuatum*	Intestine	Mexico	Pérez-Ponce de León et al. (1994); Pérez-Ponce de León et al. (2000)
*Chirostoma estor*	Intestine	Mexico	Osorio- Sarabia et al. (1986); Espinosa-Huerta et al. (1996); Pérez-Ponce de León et al. (2000)
*Ctenopharyngodon idella*	Intestine	Iraq	Jassim et al. (2022)
*Cyprinella lutrensis*	Intestine	USA	Leis et al. (2016)
*Cyprinus carpio*	Intestine	Mexico	Pérez-Ponce de León et al. (1996, 2000)
		Turkey	Dede et al. (2022)
*Danio rerio*	Intestine	USA	Murray and Peterson (2015)
*Goodea atripinnis*	Intestine	Mexico	Pérez-Ponce de León et al. (2000)
*Hemiculter leucisculus*	Intestine	Iran	Pazooki et al. (2011)
*Notropis sallei*	Intestine	Mexico	Salgado-Maldonado et al. (2001)
*Paracheirodon innesi*	Liver	Czech Republic	Moravec et al. (1984)
*Perccottus glenii*	Intestine	Germany	Kvach et al. (2017)
*Pseudorasbora parva*	Intestine	Slovakia	Mihok et al. (2011)
		Poland	Czerniejewski et al. (2019)
*Pungitius pungitius*	Intestine	Russia	Mineeva and Semenov (2023)
*Puntius tetrazona*	Intestine	Czech Republic	Moravec et al. (1984)
*Rhodeus sericeus*	Intestine	Czech Republic	Dávidová et al. (2005)
*Tinca tinca*	Intestine	Germany	Schäperclaus (1992)

*Pseudocapillaria tomentosa* is a pathogenic nematode parasite that infects zebrafish (*Danio rerio*) (Kent et al., 2002, 2019a; Maley et al., 2013; Murray and Peterson, 2015; Norris et al., 2020). The significance of *P. tomentosa* lies in its exclusive identification as the sole nematode infection found in zebrafish within research facilities (Kent and Sanders, 2020). Based on data from the diagnostic service of the NIH Zebrafish International Resource Centre (ZIRC) in Eugene, Oregon, the USA, the parasite has been detected in 15% of zebrafish research facilities (Schaaf et al., 2020; Schuster et al., 2023). Despite the controlled laboratory conditions in which zebrafish are maintained, it is noteworthy that this parasitic nematode appears to be quite prevalent in zebrafish (Kent and Sanders, 2020). Nevertheless, this phenomenon can be attributed to the absence of proper measures to ensure biological security in numerous facilities, such as sourcing fish from pet stores, as well as the ability of nematodes to endure the lower concentrations of chlorine (Martins et al., 2017).

## 2 Life cycle

An infection begins when fish ingest embryonated eggs from contaminated environments (Kent et al., 2002, 2018; Collymore et al., 2014; Gaulke et al., 2016; Mineeva and Semenov, 2023). Lomankin and Trofimenko (1982) and Matthews (2004) showed that invertebrate oligochaetes (e.g. *Tubifex tubifex*) can indeed act as paratenic hosts for *P. tomentosa* and play a significant role in transmitting it in research laboratories. Nevertheless, it has been proven that infection can also occur through direct transmission even without the presence of adult worms (Kent et al., 2001; Collymore et al., 2014; Kent and Sanders, 2020). This observation reveals why *P. tomentosa* can readily propagate and pose a potential hazard in zebrafish research facilities. The eggs undergo larval development in the host intestine and become infectious approximately 5 days after being released by the female worms when exposed to a temperature of 28 °C (Martins et al., 2017). Larvae can be identified in the intestinal mucosa within days of an infection, and mature females may typically be discovered approximately 2–3 weeks after exposure (Collymore et al., 2014; Gaulke et al., 2019). According to the findings of Kent et al. (2018) and Kent and Sanders (2020), the infection can remain widespread within a fish population for an extended period after the initial infection.

## 3 Clinical signs

**Fig. 1:**
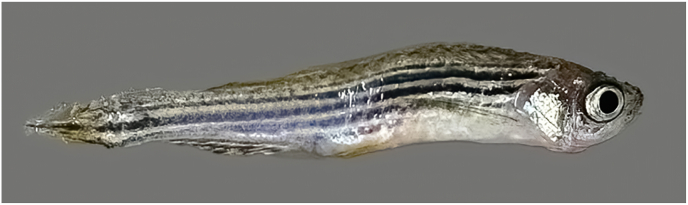
Zebrafish infected with *P. tomentosa*. Clinical signs include a humpback appearance and emaciation.

The parasite primarily triggers a chronic disease, frequently resulting in significant morbidity and mortality rates and leading to economic challenges in fish production (Mihok et al., 2011; Kent et al., 2020b; Mocho and Pereira, 2022). The most significant apparent alteration is emaciation (Kent and Sanders, 2020). In adult cyprinids, the infection can result in symptoms such as ulcers, anemia, and hemorrhage in the intestinal and digestive tracts. However, in juvenile cyprinids and zebrafish, it can lead to high mortality rates (Dede et al., 2022). In addition, there is a correlation between infections, reduced growth rate, and reproductive ability in zebrafish (Pack et al., 1995; Kent et al., 2002; Matthews, 2004). Although severe infections can result in a decline in overall health, including deformities (Fig. 1), it is not specifically stated that humpback appearances (kyphosis or lordosis) are a common symptom of *P. tomentosa* infection. Impacted fish typically display clinical symptoms such as lethargy, color darkening, and severe wasting, which is likely caused by an inability to adequately absorb nutrients (Matthews, 2004; Kent et al., 2012). Nevertheless, the fish may occasionally exhibit no apparent evidence of infection, even though it is present (Kent et al., 2012; Kent and Sanders, 2020).

## 4 Pathology

The histological analysis of the fish infected with *P. tomentosa* shows significant and widespread chronic and proliferative inflammation of the intestines and occasionally coelom (Kent et al., 2002, 2020b; Murray and Peterson, 2015; Mocho and Pereira, 2022; Schuster et al., 2023). The worms typically remain within the epithelium but can penetrate all layers of the intestine and be located within the coelom (Murray and Peterson, 2015; Gaulke et al., 2019). In addition, the presence and movement of this capillariid nematode may cause localized reactions, including epithelial hyperplasia and dysplasia (Kent et al., 2002, 2012, 2020b). Spitsbergen et al. (2000) discovered that zebrafish that were infected and exposed to a small amount of the carcinogen dimethylbenze [a]antracene had a greater occurrence of gastrointestinal tumors compared to exposed fish that were not infected. Although no distinct direct relationship has been identified between this infection and the susceptibility to intestinal neoplasms in fish thus far, the association between parasite infections and certain neoplasms has been established in higher vertebrates (e.g. the link between infection with the nematode *Spirocerca lupi* and the formation of esophageal sarcomas in dogs as well as *Schistosoma haematobium* and bladder cancer in humans) (Hicks et al., 1977; Bailey, 1979). Thamavit et al. (1996) suggested that parasites typically serve as powerful boosters, rather than initiators, of neoplasia. Infection with *P. tomentosa* causes intestinal inflammation, which can be regarded as a significant factor in promoting the development of intestinal neoplasms (Kent et al., 2002; Spitsbergen et al., 2012; Gaulke et al., 2019; Kent and Sanders, 2020; Schuster et al., 2023). Furthermore, it has been shown that intestinal inflammation can have significant effects on the composition and diversity of the gut microbiota in zebrafish (Xie et al., 2021; Liu et al., 2023). The presence of inflammation caused by parasitic infections results in a reduction in microbial diversity and an increase in the population of specific bacteria that are commonly linked to inflammatory conditions (Gaulke et al., 2019). This supports the hypothesis that parasitic infections have a significant influence on host microbial communities.

## 5 Diagnosis

**Fig. 2:**
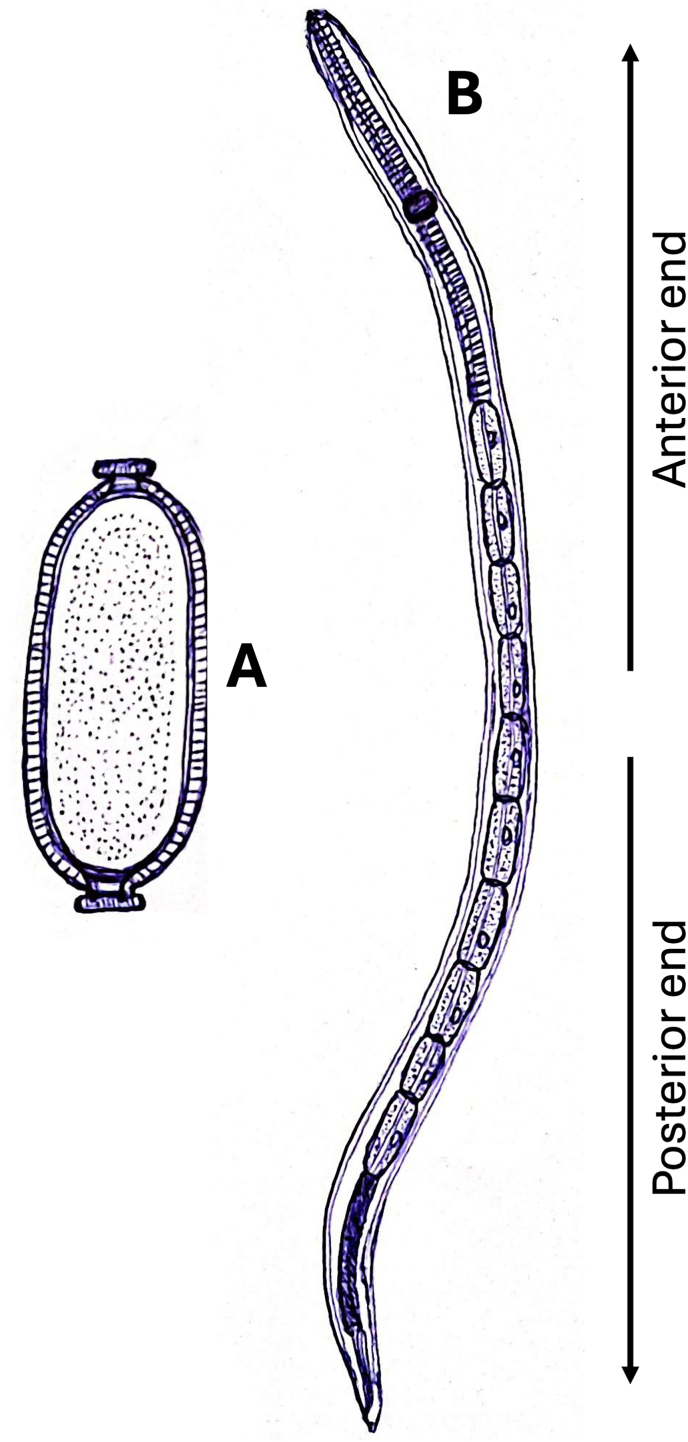
*Pseudocapillaria tomentosa*. **A** Double-operculated barrel-shaped egg with a length of 40–65 μm (Kent et al., 2002; Murray and Peterson, 2015). **B** Larva with a length range of 390–1880 μm (Moravec et al., 1984; Dávidová et al., 2005).

Diagnosing *P. tomentosa* infections can generally be relatively simple and is typically accomplished by detecting the eggs in the tank water, visualizing the worms/eggs in wet mount preparations or histological sections of the mucosal epithelium of the intestine (Kent and Sanders, 2020). However, even mild infections, in which eggs or parasites are not easily detected, might be associated with significant degenerative changes in the proximal intestinal mucosa (Kent et al., 2012). *Pseudocapillaria tomentosa* is morphologically characterized by its slender and elongated body (Fig. 2), which can reach up to 12 mm in length (Košuth, 1997; Mocho and Pereira, 2022). Male individuals, who are of smaller size compared to females, possess a specialized reproductive chitinous structures called spicules at the posterior end, which are utilized during the process of copulation (Moravec, 1982; Košuth, 1997; Dede et al., 2022). The anterior region of adult worm displays specialized structures in the esophagus region known as “stichosomes”, characterized by a clearly visible pattern of bands (Kent and Sanders, 2020). The presence of alternating dark and light coloration of the stichocysts is a distinctive characteristic of *P. tomentosa* (Kent et al., 2002). This parasitic nematode can easily be differentiated from *Pseudocapillaria nannupensis* by a distinctively expanded and folded structure of the proximal spicule rim and by having a vulval opening further to the esophago-intestinal junction (Moravec, 2001; Hobbs and Hassan, 2010).

**Fig. 3:**
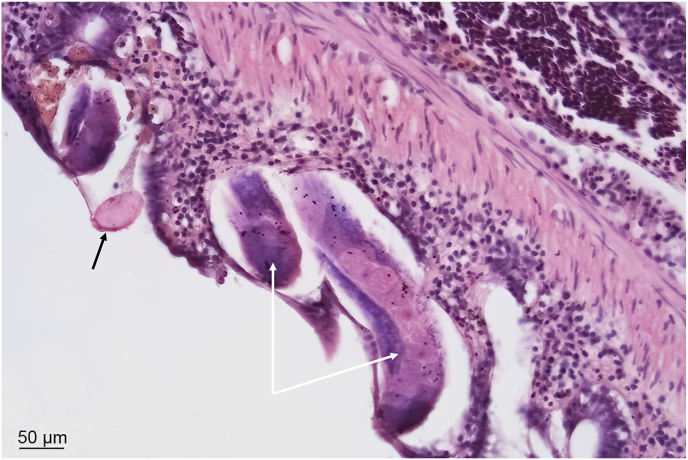
Haematoxylin & Eosin (H&E)-stained section of the intestine from zebrafish (*Danio rerio*) infected with *Pseudocapillaria tomentosa* (*white arrows*). An egg with bipolar plugs (*black arrow*) is evident in the intestinal lumen. Inflammatory cells are surrounding the damaged tissue containing the worms.

Capillariid nematodes can also be identified by distinguishing the double-operculated, barrel-shaped eggs (ranging 30–60 μm in size) in wet mount preparations or histological sections of the intestine (Fig. 3). Detection of eggs in fecal matter or sediment indicates infection. However, definitive species identification requires detailed morphological examination and molecular analyses (Ramras, 2018; Kent and Sanders, 2020; Kent et al., 2020a). The characteristic eggs can be readily differentiated from those of other parasitic or non-parasitic organisms. Various techniques can be employed to identify their presence in sediment and fecal matter, as outlined by Murray and Peterson (2015) and Kent et al. (2020a). Sugar centrifugation techniques can be employed to isolate and identify eggs of *P. tomentosa* in feces and sediment collected from zebrafish tanks (Murray and Peterson, 2015; Martins et al., 2017; Kent et al., 2020a).

PCR-based tests are increasingly used for detecting fish pathogens in aquaculture due to their high sensitivity, particularly for pathogens that do not reproduce well *in vitro*, such as many fish parasites (Adams and Thompson, 2011). These tests can detect early developmental stages that may not be easily observable under a microscope due to their small size or less distinct morphological characteristics (Schuster et al., 2023). Early detection of obligatory parasitic nematodes (e.g. *P. tomentosa*) is crucial to prevent their spread, particularly in zebrafish research facilities (Adams and Thompson, 2011; Murray et al., 2016; Schuster et al., 2023). Real-time PCR (qPCR) and digital PCR (dPCR) are effective molecular diagnostic assays for the sensitive and specific screening of *P. tomentosa* in zebrafish populations (Kent and Sanders, 2020; Norris et al., 2020).

## 6 Treatment and management

Efficient management of *P. tomentosa* necessitates the implementation of both treatment and preventive approaches; anthelmintic treatments, such as fenbendazole, levamisole, and mebendazole, have proven effective in eradicating the nematodes. However, caution should be used when treating fish with mebendazole, as it may be embryotoxic and teratogenic (Kent et al., 2002). These medications are typically provided to animals through medicated feed or by applying them as bath treatments. The initial documentation of the treatment of *P. tomentosa* in zebrafish was conducted by Pack et al. (1995). In this study, the fish were subjected to a combination of trichlorfon and mebendazole, which were administered in the form of water-soluble tablets (Kent et al., 2002). Macrocyclic lactones with anti-nematode properties have also been found to be effective in treating the infection, as reported by Kent et al. (2019a, 2020b). Kent and Sanders (2020) provided evidence that both emamectin and ivermectin were effective in significantly reducing nematode infections. However, it was found that ivermectin had adverse toxic effects, as reported by Collymore et al. (2014). Emamectin is efficacious when administered orally or as a bath treatment (Kent et al., 2020b). Kent et al. (2019a) demonstrated that administering emamectin at a dosage of 0.35 mg/kg/d for a duration of two weeks can yield substantial efficacy without any apparent adverse effects. In addition, Maley et al. (2013) administered fenbendazole orally by adding the drug at a concentration of 2 mg/l to artemia cultures, which were subsequently used as food for the fish, to treat the infection. Samaee (2015) conducted an experiment where various drugs were introduced into water and found that ivermectin, fenbendazole, albendazole, and mebendazole were all effective. Additional anthelminthic medications (e.g. levamisole), which have been examined in different fish species affected by nematode infections, also appear to be relatively effective for treating this particular infection. Nevertheless, according to reports, levamisole was found to be ineffective in treating *P. tomentosa* in zebrafish, and the administration of levamisole to brood stock zebrafish resulted in infertility (D. Weaver, personal communication).

Preventive measures primarily focus on maintaining optimal water quality, reducing fish density, and implementing strict quarantine protocols for new fish. In addition, regular monitoring and prompt intervention are essential to control the spread of the parasite and minimize its impact. Eliminating eggs of *P. tomentosa* in the system environment is a key control method, though it can be challenging in large recirculating systems (Kent and Sanders, 2020). Effective elimination can be achieved by exposing the eggs to chlorine (3000–6000 ppm) for 10 min or to heat treatments of 45–50 °C for 1 h or 40 °C for at least 8 h (Martins et al., 2017). Additionally, UV radiation (50–300 mW/cm^2^), povidone-iodine (200 ppm for 1 h), and desiccation (for at least 1 h) can also be effective (Kent et al., 2019b). Typically, if fish do not possess significant value, the most suitable course of action would be to eliminate the infected population and disinfect the system (Kent et al., 2002; Matthews, 2004). In addition, it is strongly advised to avoid using oligochaete worms as live feed as it represents a potential source of infection as these worms have been suggested to harbor the infective larval stages of *P. tomentosa* and may therefore act as the intermediate hosts for the parasite (Kent et al., 2002; Kent and Sanders, 2020; Dede et al., 2022). Furthermore, implementation of rigorous quarantine measures that only enable transfer of embryos that have been bleached (Matthews, 2004; Ramras, 2018) and conducting regular screenings of the fish to monitor possible infections would be recommended to avoid the infection (Kent et al., 2002). Moreover, integrating quantitative PCR (qPCR) assays into routine health monitoring programmes can directly aid in the elimination of specific pathogens, such as *P. tomentosa*, from zebrafish facilities at a relatively low cost (Schuster et al., 2023).

## 7 Immune modulation and adaptations in host-parasite interactions

The study of host-parasite interactions in fish has greatly enhanced our comprehension of the complex relationships between hosts and their parasites. Research has examined the impact of parasites on the health, behavior, and structure of fish populations, and has explored the ecological and evolutionary patterns of interactions between hosts and parasites. Parasites negatively impact the health of fish, potentially resulting in fatal or long-lasting diseases (Rahmati-Holasoo et al., 2023, 2024). They apply strong selective pressures on fish populations, which leads to evolutionary adaptations in both hosts and parasites (Barber et al., 2000). Furthermore, parasites can indirectly affect fish by compromising their immune system, thereby increasing their vulnerability to other diseases (Poulin, 2010; Preston and Johnson, 2010). The evolutionary dynamics of interactions between parasites and zebrafish are significant, as the ongoing co-evolutionary arms race between these two organisms leads to adaptive changes. When zebrafish populations are exposed to high levels of parasitism, they may develop stronger immune responses or adapt their behavior to reduce the risk of infection. The various selective pressures can result in a wide range of effects, impacting the genetic composition and ability to withstand challenges of zebrafish populations over time (Medel et al., 2010; Stevens, 2013).

In recent years, the zebrafish has emerged as a novel vertebrate model for studying hematopoiesis and immunology. This viable living model provides the potential to investigate the developmental immunity and associated host-microbe interactions at both the molecular and cellular levels (Galindo-Villegas, 2016). Zebrafish have a strong innate immune system, which includes pattern recognition receptors (PRRs) like Toll-like receptors (TLRs) that can identify pathogen-associated molecular patterns (PAMPs). When PAMPs are detected, the immune system is activated and produces inflammatory cytokines such as Il-1β, Tnf-α, and Ifn-γ, which play a significant role in controlling immune functions and recruiting phagocytic cells, such as macrophages and neutrophils, to the site of infection (Miao et al., 2021). Speirs et al. (2024) revealed how neutrophils and macrophages respond to immune challenges and elucidated the functional roles and dynamics of these cells in response to infections, yielding a more profound comprehension of the innate immune mechanisms in zebrafish. This study highlighted the significance of these immune cells in recognizing and reacting to pathogens, thereby strengthening the findings of Miao et al. (2021) regarding the essential roles of neutrophils and macrophages in the immune system of zebrafish.

According to Lo (2009), who examined the impact of *P. tomentosa* infection on cytokine levels in zebrafish, infected zebrafish exhibit markedly elevated expression of Tnf-α and Ifn-γ relative to uninfected fish, indicating a vigorous inflammatory response aimed at controlling the parasite. This increase is associated with the immune system’s endeavor to restrict parasite proliferation and harm, potentially resulting in inflammatory side effects within the host tissues. The discussion of eosinophils in zebrafish immunology is fundamentally dependent on the fact that both the innate and adaptive immune systems exhibit significant conservation of gene function, humoral factors, and effector cell lineages from zebrafish to mammals (Traver et al., 2003). Molecular investigations by Balla et al. (2010), Zhu et al. (2013), and Gratacap and Wheeler (2014) indicate that zebrafish gata-2 cells degranulate and release peroxidase upon exposure to helminth extract. Prolonged exposure to helminth-related allergens leads to significant eosinophilia, indicating that eosinophilic reactions to allergens have been conserved over evolution. Interestingly, the infection of adult zebrafish with *P. tomentosa* results in substantial elevations in eosinophil counts within the intestine. These findings support a preserved function of eosinophils in the reaction to helminth antigens or infections and introduce a novel model to enhance comprehension of how parasitic worms stimulate, co-opt, or evade the vertebrate immune response.

In addition to innate immunity, zebrafish adaptive immunity encompasses T and B cells, which play a vital role in specific antibody responses and T cell activation against parasites (Miao et al., 2021). Potential targets for improving resistance have been identified as specific immune pathways, including the JAK/STAT and NF-κB pathways. The JAK/STAT pathway plays an essential role in the transmission of signals from cytokines and the regulation of immune responses. It promotes the production of cytokines and strengthens the host’s defense against parasites. Similarly, by modulating the NF-κB pathway, the expression of inflammatory cytokines is increased, which enhances the host’s capacity for resistance against parasitic infections. Activating the complement system furthermore facilitates opsonization and elimination of parasites (Ivashkiv, 2012; Owen et al., 2019). While the innate immune response, which includes the generation of cytokines and the recruitment of immune cells, plays a key role in the primary protection against parasitic infections, the adaptive response and specific signaling pathways provide supplementary levels of defense and potential opportunities for improving resistance. Gaining knowledge about these interactions and the strategies parasites employ to avoid immune responses can contribute to the creation of more efficient approaches to combat parasitic infections.

## 8 Microbiota dynamics in zebrafish: shaping host-parasite relationships

The influence of microbiota on susceptibility to parasitic infections has received significant attention, as studies have demonstrated that diverse and balanced gut microbiota can enhance resistance to such infections in zebrafish. Specific bacterial strains present in the microbiota have the ability to regulate immune responses, which may enhance the host’s ability to defend itself against infections (Leung et al., 2018; Ulusan Bagci and Caner, 2022). Probiotic-based interventions are currently being studied to determine their effectiveness in enhancing gut health and resistance to parasites. This is achieved by boosting mucosal immunity and modifying the composition of the microbiota. These probiotics have the ability to enhance the protective function of barriers, and regulate immune responses, offering a promising approach to preventing parasitic infections (Leung et al., 2018; Xia et al., 2022). Supporting these findings, Li et al. (2022) explored the functions and influencing factors of the zebrafish gut microbiota, emphasizing its essential role in maintaining host health and modulating immune responses. This study revealed that gut microbiota is not only critical for nutrient absorption and metabolism but also plays a significant role in immune regulation and protection against pathogens. It also demonstrated that various factors, including diet, environment, and genetics, affect the composition and functionality of the gut microbiota, which in turn impacts the host’s susceptibility to infections. Furthermore, Colin et al. (2022) investigated the association between fish gut microbiota dysbiosis and helminths parasitism. Their study found that dysbiosis of the gut microbiota, characterized by an imbalance in microbial composition, is closely associated with helminth infections rather than exposure to polycyclic aromatic hydrocarbons (PAHs) at environmentally relevant concentrations. This research underscores the significant role that gut microbiota plays in the susceptibility to parasitic infections, highlighting that an imbalanced microbiota can predispose fish to parasitic infections, while a well-balanced microbiota enhances resistance.

Studies on the parasite *P. tomentosa* have provided deeper insights into the intricate interactions among zebrafish, gut microbiota, gut metabolites and intestinal helminth parasitic infections. These investigations can reveal histopathological changes and immune responses that occur during infection, providing a clearer understanding of the host’s defense mechanisms against parasites (Gaulke et al., 2019). Hammer et al. (2024) discovered that a substantial disparity in parasite infection burden in zebrafish correlates with the composition of the gut microbiome. Statistical mediation analysis was employed to identify a group of gut microbes whose relative abundance elucidates the relationship between gut metabolites and infection outcomes. A significant finding was the identification of salicylaldehyde, a compound presumably synthesized by the gut bacteria *Pelomonas*, as a strong anthelmintic that suppresses the hatching of *P. tomentosa* eggs both *in vitro* and *in vivo*. The research also identified N-acylethanolamines, a category of signaling molecules, in the correlation between microbiota and parasite infection burden. The findings indicated that the zebrafish gut microbiome metabolically influences intestinal helminth parasite infection outcomes and uncovers new microbiome-derived anthelmintic medication leads. Despite the research undertaken in this field, there are still deficiencies in comprehending the full spectrum of immune responses throughout various stages of development, as well as the ways in which these responses interact with environmental and microbiome factors. Additional research is required to gain a comprehensive understanding of the exact mechanisms through which certain immune pathways and microbiota impact resistance, as well as the long-term effects of administering probiotics on the immune system and overall health of zebrafish. In addition, investigations are needed to explore the relationship between the genetic composition of the host and the microbiota in determining vulnerability to parasitic infections. These findings would not only deepen our comprehension of zebrafish immunity but also have broader implications for developing novel strategies to enhance fish health in aquaculture and wild fish populations.

The evolutionary related *Trichuris trichiura*, commonly known as the human whipworm, infects nearly one billion individuals globally, resulting in intestinal tissue destruction, hemorrhage, and anemia following its invasion of the intestinal epithelium (Norhayati et al., 2003). Specific helminth species, including *Trichuris suis* and *Necator americanus*, have been examined for their potential therapeutic advantages in the treatment of autoimmune and inflammatory diseases; however, no *Capillaria* species are recognized for the treatment of human diseases to date (Weinstock and Elliott, 2013; Jenkins et al., 2021), introducing a new dimension to the *P. tomentosa*-zebrafish model. The concept of employing helminths for therapeutic purposes is founded on the immunomodulatory effects some parasites can have on their hosts, potentially alleviating symptoms in conditions marked by an excessive immune response, such as inflammatory bowel disease (IBD) and multiple sclerosis (MS). This conceptual framework presents an exciting opportunity to explore the *P. tomentosa*-zebrafish model as a platform for studying helminth-induced immunoregulation. Zebrafish possess a highly conserved vertebrate immune system, transparent embryos and larvae, and are amenable to high-throughput genetic and imaging techniques (Renshaw and Trede, 2012; Dee et al., 2016). These features make them ideal for real-time *in vivo* exploration of host-parasite interactions.

## 9 Recent advances and research directions

Recent advances in the study of *P. tomentosa* have greatly improved our understanding of its biology, pathology, and control strategies. One significant advancement has been the development of molecular diagnostics, particularly qPCR assays, which provide high sensitivity and specificity for early detection of *P. tomentosa* at low infection levels. This enables faster interventions and more effective management of parasite outbreaks in fish facilities (Norris et al., 2020; Schuster et al., 2023). Environmental and chemical control strategies have been optimized, demonstrating the efficacy of treatments such as chlorine exposure, heat treatments, UV radiation, povidone-iodine, and desiccation in eliminating parasite eggs. Alternative biological control methods are gaining popularity nowadays, with a focus on using natural predators, parasites, or competitive exclusion by non-pathogenic microorganisms to reduce the need for chemical treatments. To prevent the introduction and spread of *P. tomentosa*, strict quarantine and biosecurity measures have to be implemented, such as embryo bleaching and regular screenings. The concept of integrated pest management (IPM) is being used, which combines environmental management, chemical treatments, biological control, and genetic approaches to create a sustainable control programme. Collaborative research efforts and data sharing between laboratories and institutions have expedited advancement, allowing for the exchange of knowledge and resources and driving innovation. However, there is a need for more comprehensive research on the development of effective management and mitigation strategies to control parasitic infections in both aquaculture and perhaps wild fish populations. These multidisciplinary approaches aim to reduce the impact of parasites while lowering environmental and economic costs, ultimately improving the health and productivity of fish populations in sustainable aquaculture.

## 10 Conclusion

In conclusion, effective managing host-parasite interactions necessitates a comprehensive understanding of immunological mechanisms, microbiome impacts, and practical control strategies. Integrating these concepts not only improves parasitic infection management but also promotes environmentally responsible practices. By utilizing interdisciplinary research, we can foster resilient fish populations, reduce reliance on chemical treatments, and assure long-term sustainability in aquaculture.

## CRediT authorship contribution statement

**Amin Marandi:** Conceptualization, Writing – original draft, Writing – review & editing. **Anne Majgaard Jensen:** Writing – review & editing. **Louise von Gersdorff Jørgensen:** Conceptualization, Writing – review & editing, Supervision, Project administration, Funding acquisition.

## Ethical approval

Not applicable.

## Data availability

The data supporting the conclusions of this article are included within the article.

## Funding

Co-funded by the 10.13039/501100000780European Union’s Horizon Europe programme, project EUPAHW (number 101136346). Views and opinions expressed are, however, those of the author(s) only and do not necessarily reflect those of the European Union or REA. Neither the European Union nor REA can be held responsible for them.

## Declaration of competing interests

The authors declare that they have no known competing financial interests or personal relationships that could have appeared to influence the work reported in this paper.

## Data Availability

The data supporting the conclusions of this article are included within the article.
